# Caloric restriction stabilizes body weight and accelerates behavioral recovery in aged rats after focal ischemia

**DOI:** 10.1111/acel.12678

**Published:** 2017-09-29

**Authors:** Ovidiu Ciobanu, Raluca Elena Sandu, Adrian Tudor Balseanu, Alexandra Zavaleanu, Andrei Gresita, Eugen Bogdan Petcu, Adriana Uzoni, Aurel Popa‐Wagner

**Affiliations:** ^1^ University Psychiatric Center Basel Switzerland; ^2^ University of Medicine and Pharmacy Craiova Neurobiology of Aging Group Craiova Romania; ^3^ Griffith University School of Medicine Gold Coast Campus Gold Coast Qld 4222 Australia; ^4^ Department of Psychiatry Aging & Psychiatric Disorders Group University of Medicine Rostock Rostock Germany

**Keywords:** aging, behavior, body weight, calorie restriction, neuroprotection, stroke, transcriptomics

## Abstract

Obesity and hyperinsulinemia are risk factors for stroke. We tested the hypothesis that caloric restriction, which reduces the incidence of age‐related obesity and metabolic syndrome, may represent an efficient and cost‐effective strategy for preventing stroke and its devastating consequences. To this end, we placed aged, obese Sprague‐Dawley aged rats on a calorie‐restricted diet for 8 weeks prior to the experimental infarction. Stroke in this animal model caused a progressive decrease in weight that reached a minimum at day 6 for the young rats, and at day 10 for the aged, *ad libitum*‐fed rats. However, in aged animals that were calorie‐restricted prior to stroke, body weight did not decrease after stroke, but we noted accelerated body weight gain shortly thereafter starting at day 5 poststroke. Moreover, calorie‐restricted aged animals showed improved behavioral recovery in tasks requiring complex sensorimotor skills, or in tasks requiring cutaneous sensitivity and sensorimotor integration or spatial memory. Likewise, calorie‐restricted aged rats showed significant poststroke increases in serum glucose, insulin, and IGF1 levels, as well as CR‐specific changes in the expression of gene transcripts involved in glycogen metabolism, IGF signaling, apoptosis, arteriogenesis, and hypoxia. In conclusion, our study shows that recovery from stroke is enhanced in aged rats by a dietary regimen that reduces body weight prior to infarct.

## Introduction

Stroke represents a potentially lethal condition with devastating effects for patients and their families. One of the most important risk factors for stroke is represented by obesity. At the present time, numerous studies conducted in the Western World have recorded an ‘obesity epidemic’. The American Heart Association (AHA) guidelines indicate that obesity and body fat distribution as well as poor diet and physical inactivity are well‐documented risk factors, which can be adjusted by an adequate lifestyle (Goldstein *et al*., 2011).

Clinical studies have indicated that obesity is characterized by a body mass index (BMI) of at least 30 kg m^−2^, while those above this limit are labeled as overweight. More importantly, it seems that for overweight and obese patients, every 5 kg m^−2^ is associated with 40% increased mortality if the patient develops any type of stroke (Goldstein *et al*., 2011). Therefore, at the present time, AHA recommends weight reduction in those overweight and obese to reduce the risk of stroke. Recently, the same medical organization recommends a simple rule termed ‘Life's simple 7′ for preventing cardiovascular events including stroke. This includes maintaining normal weight, glucose, blood pressure, and cholesterol, being physically active, nonsmoking, and eating a healthy nutritious diet. However, at least four criteria must be met for a good outcome after stroke (Lin *et al*., 2015). In fact, obesity is never described as a single abnormality as it by itself leads to other pathological complications such as hypertension, diabetes, and hypercholesterolemia (Towfighi & Ovbiagele, 2009; Strazzullo *et al*., 2010; Li *et al*., 2013).

Strazzullo *et al*. (2010) conducted a database analysis of 25 stroke studies (more than 2 million patients and more than 30 000 events) that revealed the association of obesity with both hemorrhagic and ischemic stroke independent of other stroke risk factors (Strazzullo *et al*., 2010). Paradoxically, after the acute event, the highest mortality risk was described in young patients with increased BMI, while in obese elderly patients the risk decreased in a linear fashion. The authors claim that elderly obesity may induce a protective effect (Towfighi & Ovbiagele, 2009). However, this finding is poorly understood and is yet to be verified in more experimental and clinical studies.

Other studies have mentioned that obesity could represent a state of chronic inflammation, which in a selected group of patients promotes specific vascular events. In fact, obese female patients in apparent state of health are at risk of developing an ‘obesity‐related pulmonary embolism’ (ORPE), which has a worse prognosis and is associated with more complications than the ‘non‐obesity‐related pulmonary embolism’ (NORPE). In keeping with the above, other studies have shown that thrombolytic therapy is less successful in obese patients. It was reported that patients with ischemic stroke and metabolic syndrome associated with abdominal obesity do not respond well to *iv* administration of tPA. The authors recorded by radiological methods (transcranial Doppler) a lack of recanalization at 24 hours after tPA administration (Arenillas *et al*., 2008; Deguchi *et al*., 2012). We can only speculate that at molecular level, various inflammatory factors released in the context of obesity interfere with the action of tPA preventing thrombolysis.

It has been suggested that manipulation of various factors secreted poststroke could improve, at least theoretically, the outcome poststroke (Pradillo *et al*., 2016). An example is represented by the inhibition of interleukin‐1 (IL‐1), a pro‐inflammatory cytokine secreted in stroke patients, after therapy with interleukin‐1 receptor antagonist (IL‐1Ra). This factor promotes recuperation after ischemic stroke in overweight Wistar rats enhancing the development of neuroblasts and newly poststroke formed neurons (Pradillo *et al*., 2016).

Moreover, other studies suggest that apart from molecular factors secreted in the setting of obesity and ischemic stroke, the integrity of the blood–brain barrier is affected in obese subjects. Thus, it was reported that at 4 and 24 h after the ischemic event, the obese mice have a significant blood–barrier breakdown characterized by an increased amount of endothelial vesicles, suggesting vascular damage (Haley & Lawrence, 2016). However, it is not clear which are the factors that modulate the blood–brain barrier damage.

Currently, the determinant role of overweight‐obesity as a risk factor for stroke is widely accepted. Therefore, there is an increasing interest to investigate the mechanisms by which calorie restriction could act as a neuroprotective method in obese subjects after stroke (Arumugam *et al*., 2010). Moreover, it has been shown that caloric restriction in a murine model provides protection against myocardial ischemia via significant elevation of phosphorylated GSK3‐beta and Akt while decreasing the levels of cyclo‐oxygenase IV, cytochrome C, and Pgc‐1 (Noyan *et al*., 2015).

Caloric restriction is associated with several physiological changes such as abolition of the sexual reproductive functions, decreased body temperature while in parallel there is a reduction in free insulin and glucose, indicating a decrease in white adipose tissue mass. In addition, in parallel with a change from carbohydrate to adipose metabolism, it seems that several molecular pathways are activated by caloric restriction: the target of rapamycin (TOR), sirtuin, adenosine monophosphate‐activated kinase, and insulin‐like growth factor (IGF‐1) (Speakman & Mitchell, 2011).

However, at the present time, we do not have a clear understanding of the molecular pathways and factors determined by the aging process or obesity and how they interfere with brain damage and recuperation after the acute event. In this context, numerous groups have conducted research on the beneficial effects of calorie restriction on the pathophysiology of aged subjects. Unfortunately, except a reduction in neuroinflammation (Arumugam *et al*., 2010), little is known about the effects of calorie restriction on behavioral recovery and molecular parameters after stroke in aged comorbid animal models.

In this context, we have embarked on a thorough characterization of the caloric restriction effects on behavioral, genetic, and biochemical factors in an obese, aged rat model of ischemic stroke.

## Results

**Figure 1:**
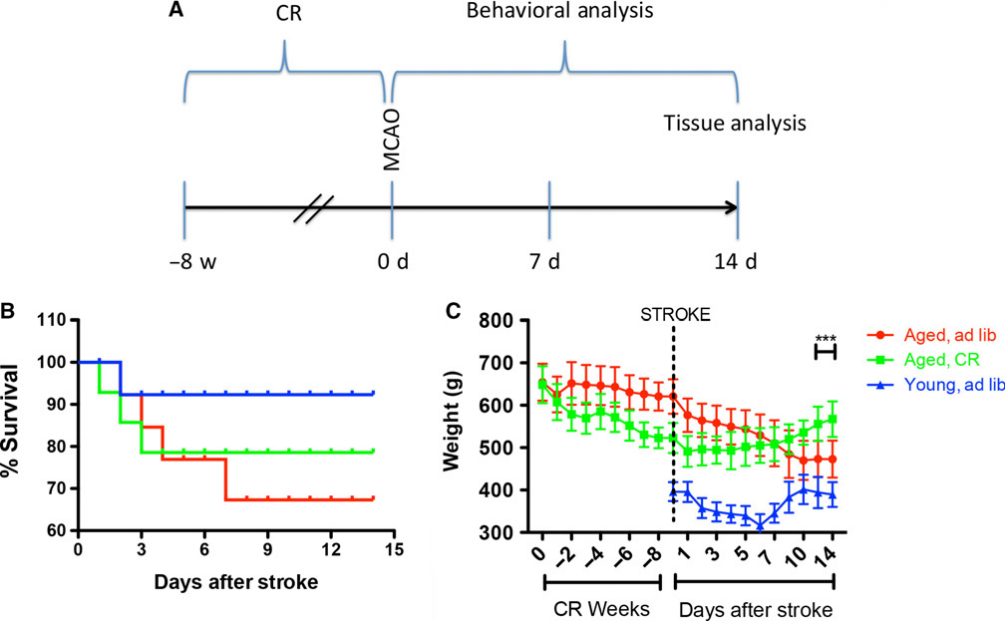
(A) Experimental design. (B) The mortality rate was 8% for the young rats, 22% for the CR rats, and 32% for the aged, ad libitum‐fed rats. (C) After stroke, the body weight declined progressively in 3‐month‐old and 20‐month‐old ad libitum‐fed animals. While young rats began to regain weight by day 7, the aged *ad libitum*‐fed animals stopped losing weight by day 10 and barely recovered the presurgery weight by day 14. Nevertheless, in contrast to both young and aged *ad libitum*‐fed animals, the CR aged rats began to regain weight already by day 2 and reached presurgery levels by day 14.

For the first 3 days after the stroke, animals in all groups were fed soft pellets to facilitate nutrition, and thereafter, they were permitted to eat *ad libitum*. Following infarction, all rats had diminished performance on the first 3 days postsurgery, which was at least partially attributable to the surgical procedure itself. The mortality rate was 8% for the young rats, 22% for the calorie‐restricted (CR) rats, and 32% for the aged, ad libitum (AL)‐fed rats (Fig. 1B).

### Body weight and basal metabolic parameters before stroke

We placed aged, obese rats on a calorie‐restricted diet for 8 weeks prior to the experimental infarction. During this period, the rats lost approximately 20% of their body weight. The prestroke basic metabolic characteristics of the rats used in this study are summarized in Table 1. There was a progressive increase in the weight of the young rats by 20 months of age, which was prevented partially by CR (Fig. 1C). There was a significant effect of CR on aged animals weight (*F* = 57.18, *P* < 0.0001), and the weights changed significantly over time (*F* = 39.09, *P* < 0.0001); in addition, there was a significant CR × time interaction (*F* = 40.73, *P* < 0.0001).

**Table 1:** Body composition and basal metabolic characteristics of SD rats before stroke

	3‐month‐old	20‐month‐old, *ad lib*	20‐month‐old CR
Body weight (g)	396.25 ± 22.40	620.73 ± 40.77	523 ± 35.50*
Fat mass (g)	34.56 ± 3.99	201.77 ± 28.10	89 ± 15,24*; *N* = 7
Glucose (mmol/L)	4.13 ± 0.20	4.2 ± 0.20	4.1 ± 0.18
Insulin (μg/L)	0.54 ± 0.040	1.20 ± 0.07	0.668 ± 0.06***
FFA (mg/dL)	19.82 ± 4.29	40.62 ± 10.98	19.51 ± 7.56***
IGF‐1 (ng/mL)	1761 ± 78	1049 ± 58	825 ± 47*

Data are means ± SEM; *N* = no of animals; *P < 0.05 vs. 20‐month‐old, *ad lib;* ***P < 0.001 vs. 20‐month‐old, *ad lib*.

Calorie‐restricted had a significant effect on serum IGF1 (22% decrease; *P* < 0.05), insulin (45% decrease; *P* < 0.001), and free fatty acids (FFA) (52% decrease; *P* < 0.001) concentration in plasma. Caloric restriction also led to a significant decrease (2.2‐fold; *P* < 0.05) in total fat mass in aged animals (Table 1).

### Body weight and metabolic parameters after stroke

After stroke, the body weight declined progressively in 3‐month‐old and 20‐month‐old ad libitum‐fed animals (Fig. 1C). While young rats began to regain weight by day 7, the aged *ad libitum*‐fed animals stopped losing weight by day 10 and barely recovered the presurgery weight by day 14. Nevertheless, in contrast to both young and aged *ad libitum*‐fed animals, the CR aged rats began to regain weight already by day 2 and reached presurgery levels by day 14 (Fig. 1C). There was a significant effect of CR on animal weight (*F* = 11.36, *P* < 0.0001), and the weights changed significantly over time (*F* = 30.09, *P* < 0.0001); in addition, there was a significant treatment × time interaction (*F* = 33.23, *P* < 0.0001).

**Figure 2:**
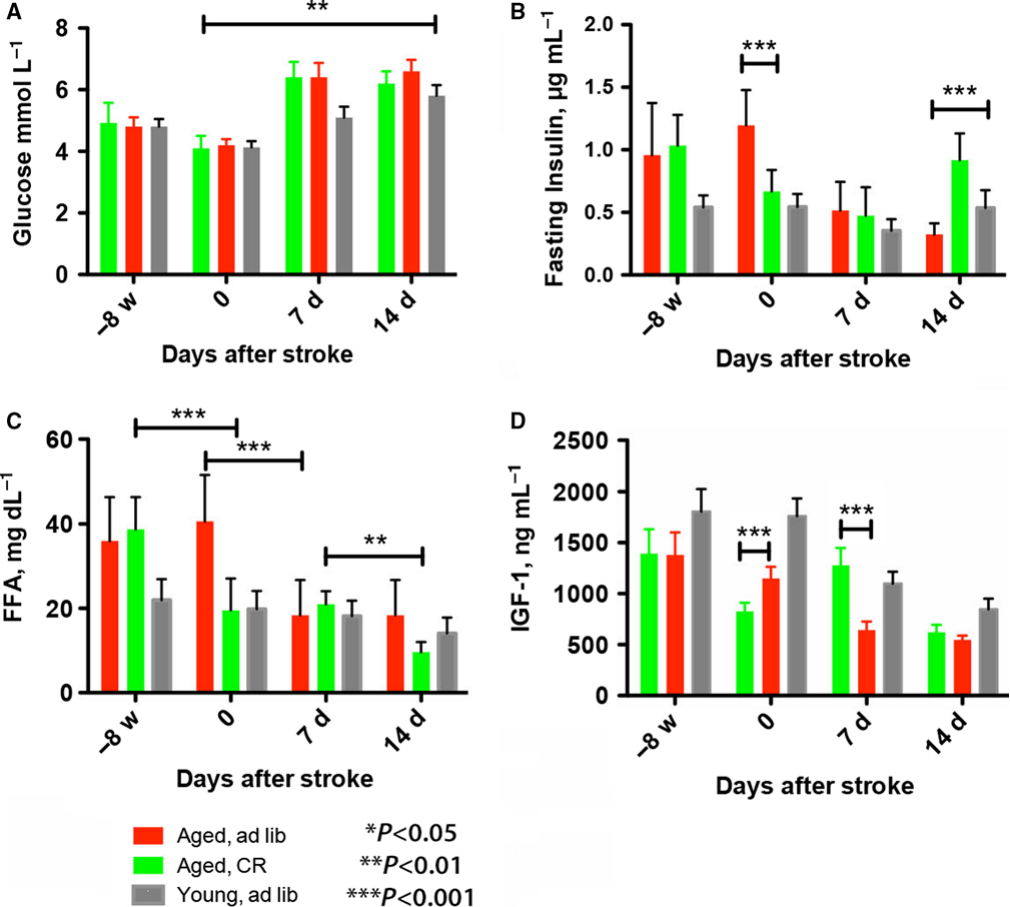
Metabolic parameters after stroke. Differences between groups were determined by two‐way ANOVA with Bonferroni *post hoc* tests at *P* ≤ 0.05 (two‐tailed). By this criterion, glucose serum levels reached a statistically significant difference by day 14 poststroke in CR vs AL animals (A). By day 14 after stroke, insulin levels significantly increased in aged CR rats as compared to the young and AL animals (B). After stroke, free fatty acid levels decreased significantly in AL animals and by day 14 also in CR animals (C). By day 7, the circulating IGF1 levels significantly decreased in AL aged animals as well as young rats, but not in CR animals (D). *P ≤ 0.05; **P ≤ 0.01; ***P ≤ 0.001.

At the end of the testing period, serum glucose levels increased significantly in all groups. There was a significant effect of CR on glucose serum concentration (*F* = 32.83, *P* < 0.0001), and the glucose levels changed significantly over time (*F* = 274.1, *P* < 0.0001); in addition, there was a significant treatment × time interaction (*F* = 12.88, *P* < 0.0001) (Fig. 2A). Fasting serum insulin was significantly decreased by calorie restriction in aged animals (Fig. 2B). By day 14 after stroke, insulin levels significantly increased in aged CR rats as compared to the young (1.7‐fold) and AL aged (2.8‐fold) animals (Fig. 2B). There was a significant effect of CR on insulin serum concentration (*F* = 206.7, *P* < 0.0001), and the insulin levels changed significantly over time (*F* = 22.76, *P* < 0.0001); in addition, there was a significant treatment × time interaction (*F* = 31.78, *P* < 0.0001). By day 7, the circulating IGF1 levels significantly decreased in AL aged animals (twofold) as well as young rats, but not in CR animals (Fig. 2D). There was a significant effect of CR on IGF1 serum concentration (*F* = 14.34, *P* < 0.0002), and the IGF1 levels changed significantly over time (*F* = 152.9, *P* < 0.0001); in addition, there was a significant treatment × time interaction (*F* = 56.24, *P* < 0.0001). After stroke, free fatty acid (FFA) levels decreased significantly in *ad libitum*‐fed animals and by day 14 also in CR (1.9‐fold) animals (Fig. 2C). There was a significant effect of CR on FFA serum concentration (*F* = 16.76, *P* < 0.0001), and the FFA serum levels changed significantly over time (*F* = 85.17, *P* < 0.0001); in addition, there was a significant treatment × time interaction (*F* = 22.09, *P* < 0.0001).

#### Behavior

##### The beam‐walking task

**Figure 3:**
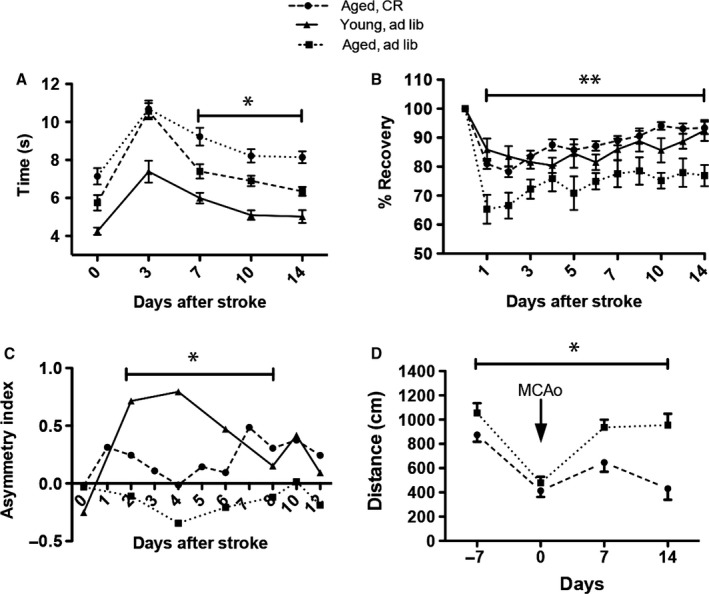
To evaluate changes in neurological function associated with ischemia, the rats were subjected to a variety of locomotor (rotating pole, A; inclined plane, B), cutaneous sensitivity and sensorimotor integration (C), learning and memory (water maze, D) tests before and after surgery. Results obtained before surgery for the inclined plane were used to define 100% functionality before cerebral ischemia and functional recovery were expressed as percentage recovery relative to the presurgery baseline. *P ≤ 0.05; **P ≤ 0.001.

Because successful performance on the rotating rod requires complex sensorimotor skills, the after effects of the surgical procedure itself were evident in the first 3 days poststroke, at which time all animals, including the young controls, had difficulty traversing the rotating cylinder. After an abrupt decline in performance on the rotarod at day 3 poststroke, young rats began improvement and almost recovered by day 14 (Fig. 3A). In contrast, the aged *ad* libitum‐fed rats never recovered fully (Fig. 3A). This was not recorded in aged CR rats, in which recovery performance levels were better than the performance of their counterparts AL aged animals (Fig. 3A). There was a significant effect of CR on poststroke recovery in aged rats (*F* = 90, *P* < 0.0001), and the recovery improved significantly over time (*F* = 46.34, *P* < 0.0001).

##### Inclined plane

In aged rats, stroke severely impaired performance on the inclined plane test, and the *ad libitum*‐fed aged rats recovered to a limited extent during the study period (Fig. 3B). Caloric restriction was effective throughout the study period, and allowed aged rats to recover to the levels attained by young rats (Fig. 3B). There was a significant effect of CR on poststroke recovery (*F* = 11.36, *P* < 0.0001), and the weights changed significantly over time (*F* = 30.09, *P* < 0.0001); in addition, there was a significant treatment × time interaction (*F* = 33.23, *P* < 0.0001).

##### The adhesive tape removal test

The asymmetry index test probes for differences between forelimbs in cutaneous sensitivity and sensorimotor integration after stroke. As compared to preoperative, trained animals, MCAO animals demonstrated a marked difference in postoperative performance for the left (affected) forelimb. By day 3 poststroke, animals started recuperation and reached significant recovery of function during the entire testing period in the CR‐fed group as compared to the *ad libitum*‐fed animals albeit not to the levels attained by young rats (Fig. 3C). There was a significant effect of CR on poststroke recovery of the asymmetry index in aged animals (*F* = 98.7, *P* < 0.0001), and the asymmetry index changed significantly over time (*F* = 40.16, *P* < 0.0001); in addition, there was a significant treatment X time interaction (*F* = 62.02, *P* < 0.0001).

##### Water maze

Over the prestroke training period of 7 days, rats learned to locate and climb onto the hidden platform and performance improved significantly during this time. In all groups, the path became shorter as the training sessions progressed (Fig. 3D). Because of the skull injury, we avoided testing the animals in the first week poststroke. As previously shown, aged rats need more time to recover behaviorally after stroke than young animals (Buchhold *et al*., 2007). Consequently, the path length required to reach the platform in the third quadrant reached a maximum by day 7 poststroke. After 7 days, the animals began recovering in this test. The best recovery was seen for the CR group, which showed significant improvement of spatial reference memory between days 7 and 14 as compared to the control group (*F* = 26.7; *P* = 0.0001). Recovery of spatial reference memory also changed significantly over time (*F* = 17.85, *P* < 0.0001); in addition, there was a significant treatment × time interaction (*F* = 62.02, *P* < 0.0001; Fig. 3D).

#### Infarct volume

**Figure 4:**
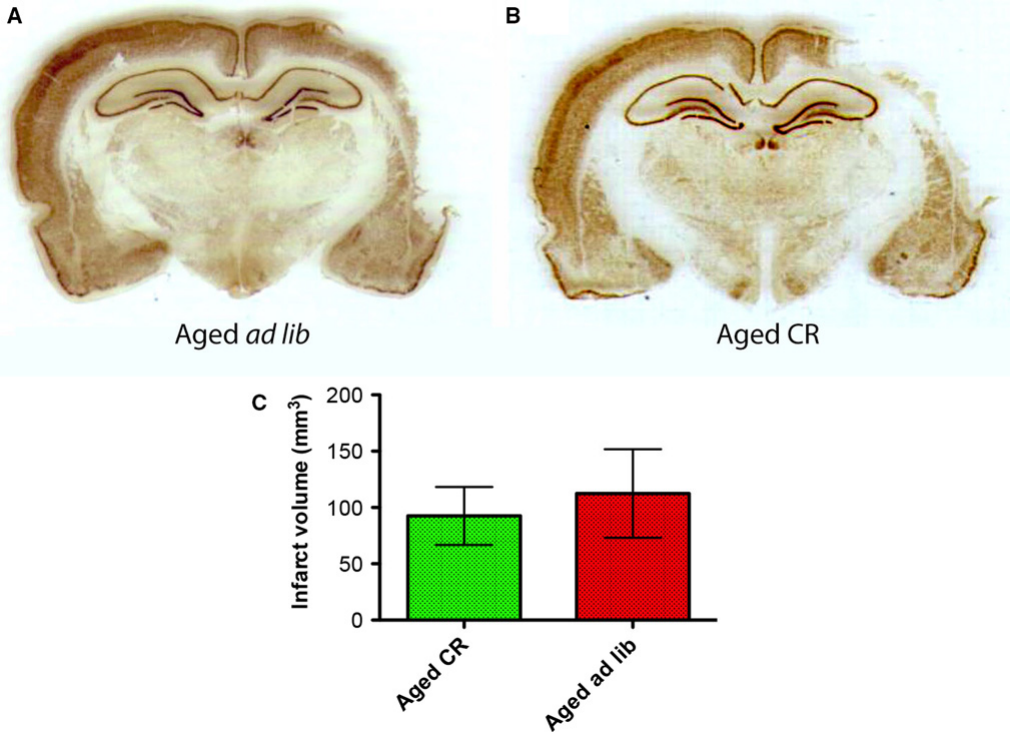
Immunohistochemical staining of the infarct area at day 14 using an anti‐NeuN antibody showed that the infarct volumes were not significantly different between the *ad libitum*‐fed aged animals and aged CR rats.

Immunohistochemical staining of the infarct area at day 14 using an anti‐NeuN antibody showed that the infarct volumes were not significantly different between *ad libitum*‐fed aged animals and aged CR rats (Fig. 4).

#### Gene expression

##### Upregulated genes

**Figure 5:**
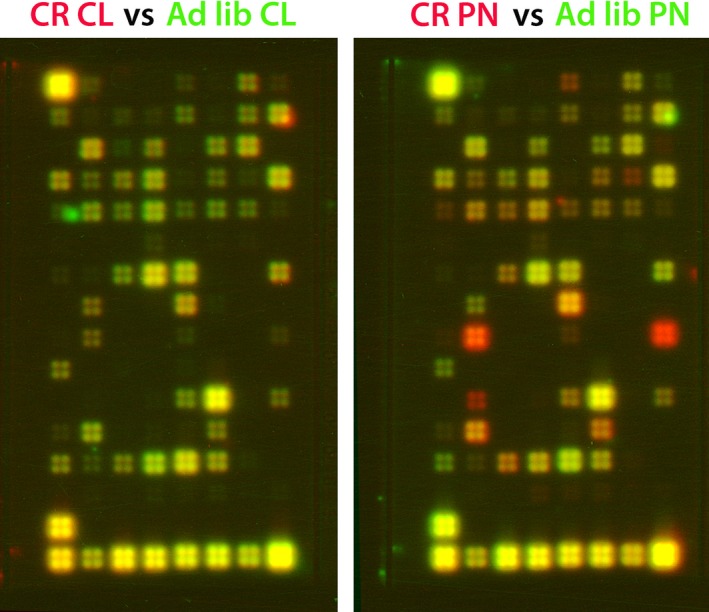
Identification of metabolic genes underlying the effect of caloric restriction on biochemical parameters and behavioral recovery after stroke using custom cDNA arrays. The gene expression fold change was expressed as the mean of two experiments. Only those genes whose expression was equal to or greater than a twofold change were considered to be differentially regulated.

Genes that were upregulated in the perilesional cortex of calorie‐restricted aged rats, as compared to the perilesional area of *ad libitum*‐fed aged rats (Fig. 5), included those associated with glycogen metabolism, IGF signaling, apoptosis, arteriogenesis, and hypoxia (Table 2). Remarkably, one of these genes, Prkaac/Prkga1, is localized to specific subcellular structures that corresponded with the expression pattern of glycogen phosphorylase and it has been associated with an increased availability of cellular energy from glycogen stores (Pirianov *et al*., 2003). The upregulated genes included genes that have a neuroprotective effect (*Igfbp3*) and genes that support angiogenesis (*Igf2*,* Mapkapk2*). The proangiogenic genes showed a 2.2‐ to 4.2‐fold increase in CR aged rats as compared to *ad libitum‐*fed aged rats. Likewise, we noted high‐level expression (2.2‐ to 6.5‐fold increases over the perilesional area of ad libitum‐fed aged rats) of *Igf2* and *Mapkapk2* that promote both endothelial precursor cell (EPC) recruitment and incorporation into the neovascular area, resulting in an enhanced angiogenesis in vivo subsequent to hypoxia and inflammation (Maeng *et al*., 2009; Limbourg *et al*., 2015).

**Table 2:** Genes whose expression was upregulated in response to calorie restriction and focal cerebral ischemia

Gen bank	Symbol	Description	Gene name	Ref. start	Ref. end	Fold change
NM_138867	Hyoul	Hypoxia upregulated 1	Cabl40/Orpl50	4386	4406	2.4 ± 0.21
NM_013010	Prkagl	Protein kinase, AMP‐activated, gamma 1	Prkaac/Prkgal	1216	1236	2.33 ± 0.22
NM_178102	Mapkapk2	MAP kinase‐activated protein kinase 2	Mapkapk2	2772	2792	2.21 ± 0.16
NM_031511	Igf2	Insulin‐like growth factor 2	IGFII/RNIGF2	4109	4129	6.55 ± 1.05
NM_012588	Igfbp3	Insulin‐like growth factor binding protein 3	IGF‐BP3	2187	2207	4.21 ± 0.88
NM_012904	Anxal	Annexin Al	Anxl/p35	1091	1111	7.89 ± 0.77

##### Downregulated genes

Downregulated genes in the perilesional cortex of the calorie‐restricted aged rats, as compared to the perilesional area of *ad libitum*‐fed aged rats, included genes involved in energy homeostasis (*Igf1r, Mapk10),* apoptosis *(Camk2 g),* vasculogenesis *(Ppp2cb),* or tissue integrity (*Pmds6, Psmc4, Pmc3, Psmb5*; Table 3).

**Table 3:** Genes whose expression was downregulated in response to calorie restriction and focal cerebral ischemia

Gen bank	Symbol	Description	Gene name	Ref. start	Ref. end	Fold change
NM_198730	Psmd6	Proteasome (prosome, macropain) 26S subunit, non‐ATPase, 6	P44sl0	968	988	2.41 ± 0.37
NM_057122	Psmc4	Proteasome (prosome, macropain) 26S subunit, ATPase, 4	Tbp7	1285	1305	2.2 ± 0.19
NM_031595	Psmc3	Proteasome (prosome, macropain) 26S subunit, ATPase 3	Psmc3	1568	1588	3.21 ± 0.47
XM_341314	Psmb5	Proteasome (prosome, macropain) subunit, beta type 5	Psmb5	594	614	2.46 ± 0.42
NM_017040	Ppp2cb	Protein phosphatase 2 (formerly 2A), catalytic subunit	Pp2a2	1507	1527	2.33 ± 0.19
NM_019129	Insl	Insulin 1	Ins1	373	393	4.75 ± 0.52
NM_052807	Igflr	Insulin‐like growth factor 1 receptor	IGFIRC/JTK13	4228	4248	5,21 ± 0.67
NM_012806	MapklO	Mitogen‐activated protein kinase 10	Jnk3/SAPKC	1916	1936	6.12 ± 0.89
NM_133605	Camk2 g	Calcium/calmodulin‐dependent protein kinase II gamma	Camk2 g	1669	1689	2.76 ± 0.57

Camk2 g links endoplasmic reticulum stress with Fas and mitochondrial apoptosis pathways (Timmins *et al*., 2009). Experimental studies have shown that a reduced expression of the catalytic subunit (Ppp2cb) of the protein phosphatase 2 (formerly known as 2A) promotes estrogen‐dependent inhibition of vascular smooth muscle cells (Ueda *et al*., 2013) and indirectly supports vasculogenesis. Interestingly, the lesioned cortical area of CR animals was better protected against degradation by downregulation of several components of the ubiquitin proteasome degradation system (*Pmds6, Psmc4, Pmc3, Psmb5*).

Transcripts encoding insulin and insulin‐like growth factor 1 receptor in the perilesional area of CR animals were severely downregulated (about fivefold). The insulin‐like growth factor 1 (IGF‐1) receptor is activated either by Igf1 or by Igf2 and is neuroprotective following brain injury (Werner & LeRoith, 2014).

JNK3 (encoded by the *Mapk10* gene) modulates the energy balance in response to metabolic stress. Studies conducted on hypothalamic neurons have proved that lack of JNK3 at this level induces hyperphagia (Vernia *et al*., 2016). More importantly, disruption of Mapk10 (JNK3) is neuroprotective in a mouse model of ischemia through a complex and poorly understood mechanism (Pirianov *et al*., 2006).

## Discussion

It is widely accepted that both aging and obesity are risk factors for ischemic stroke. The study of these aspects is often difficult due to the fact that aged rats are often time comorbid (Martin *et al*., 2010). We report that weight reduction, before ischemic stroke, in aged, overweight rats induced by caloric restriction led to an early regain of weight. Moreover, we are reporting a significant improvement in behavior as measured by tests that require complex sensorimotor skills, such as the rotating rod and inclined plane tasks, percutaneous sensitivity and sensorimotor integration, or spatial memory.

Weight loss after stroke is a common feature both in rodents and in human subjects and is related to impaired feeding, reduced physical activity, sympathetic activation, fever, inflammation, and metabolic imbalances due to insulin resistance, dyslipidemia, or endothelial dysfunction. As a result, accelerated muscle waste and cachexia may occur. Therefore, it can be speculated that overweight patients could have a better outcome as they can, at least theoretically, counteract all of the above (Scherbakov *et al*., 2011). However, the complications determined by obesity, including the risk for stroke, outweigh by far the benefits poststroke.

Before stroke, the CR animals were characterized by a reduction in body weight, adipose tissue mass, circulating insulin, IGF1, and free fatty acids (FFA) levels as compared to the *ad libitum*‐fed animals. Interestingly, in caloric restriction aged rats, we did not record a decrease in body weight after stroke but we noted accelerated body weight regain shortly thereafter. This contradicts the clinical observations discussed by Scherbakov *et al*. (2011) but more experiments are needed to fully understand the significance of our findings. However, in our studies, weight gain and behavioral recovery improved in animals subjected to calorie restriction. Before stroke, we noted increases in fat mass, serum free fatty acids, insulin, and IGF1 levels in *ad libitum*‐fed, aged rats that is suggestive of a state of glucose intolerance with hyperinsulinemia. Usually, glucose intolerance is associated with insulin resistance and insulin secretory defects in aging humans although other factors may contribute to the development of insulin resistance with age, such as obesity and lack of physical activity (Chang & Jeffrey, 2003). Therefore, our experiments have revealed some rather unexpected results in contrast with previously published studies suggesting that the beneficial effect of decreased fat mass on body weight recovery in CR animals is explained by a decreased insulin resistance, which is common in both aging rats (Barzilai & Gupta, 1999) and humans (Basu *et al*., 2003). Moreover, it is known that IGF‐1 drops in parallel with increasing age in mice and rat as a result of a significant decrease in protein synthesis ability (Sonntag *et al*., 1999). A recent study reported that caloric restriction reduces plasma IGF‐1 levels by 20% in mice (Berrigan *et al*., 2002).

In patients that were given an acute caloric restriction diet, it was reported a decrease in serum free IGF‐I, while the IGFBP‐1 reached high levels (Henning *et al*., 2013).

These data are in contrast with our experiments on calorie‐restricted aged rats that have showed an increase in IGF‐1 levels in the first week after stroke. However, considering the weight gain and behavioral recovery in CR aged rats, we could state that our results confirm the findings of some epidemiological studies that have noted an inverse relationship between plasma IGF‐I levels and the risk of stroke. The same trend has been observed between plasma IGF‐1 and the clinical recuperation and outcome after stroke. More specifically, the subjects with low plasma IGF‐I levels have a higher risk of stroke and have a worse prognosis after stroke than those patients with high plasma IGF‐1 (Bondanelli *et al*., 2006; Aberg *et al*., 2011; Denti *et al*., 2004).

It has also been suggested that in ischemic stroke patients, circulating IGF‐1 represents a marker of functional performance and outcome (Bondanelli *et al*., 2006). These conclusions have been subsequently confirmed by Aberg *et al*. (2011). They suggest that during the neurorehabilitation after stroke, a high serum IGF‐1 correlates with recuperation of long‐term functions (Aberg *et al*., 2011). Indeed, both hemorrhagic stroke and ischemic stroke are associated with low serum concentrations of IGF‐1 and IGFBP‐3, deficits in neuromuscular performance, and selective muscle atrophy (Silva‐Couto *et al*., 2014).

Subsequent to an acute ischemic event, IGF‐1 crosses the disrupted blood–brain barrier and can induce differentiation of neural cells, including neurons, astrocytes, oligodendrocytes, and endothelial cells, in in vitro and animal models of traumatic brain injury and thus may exert its neuroprotective effects (Mangiola *et al*., 2015). In parallel, insulin‐like growth factor binding protein‐3 (IGFBP‐3) modulates the bioavailability, transportation, and localization of insulin‐like growth factor‐I (IGF‐I), in animal stroke models especially when administered intranasally (Liu *et al*., 2001). In our model, CR rats had significantly increased serum IGF1 levels in the first week after stroke that coincided with an accelerated recovery of body weight; IGF‐1 level was further maintained at day 14 by an increase in serum insulin levels. This setting could be similar to perinatal hypoxia/ischemia, which increases cerebral vascular endothelial IGFBP3 expression. IGFBP‐3 modulates cell fate by a complex interplay between cells’ microenvironments and the presence of cellular IGFBP‐3 binding partners and growth factor receptors. However, an increased serum IGFBP3 (by 100%) in CR animals (Olivo‐Marston *et al*., 2014) could explain the elevated gene expression for IGFBP3 in the perilesional area of CR animals. Indeed, recent studies conducted 1 year after an acute ischemic stroke have indicated that IGFBP3 could represent an independent marker for functional outcome and recovery (Ebinger *et al*., 2015).

Periodic fasting promotes health by an increased insulin sensitivity, reduced blood pressure, body fat, IGF‐I, insulin, glucose, atherogenic lipids, and inflammation. Fasting also improves functional outcome in animal models of disorders that include myocardial infarction, diabetes, stroke, Alzheimer's disease, and Parkinson disease (Ravussin *et al*., 2014; Longo & Mattson, 2014). Moreover, the available data suggest that prolonged starvation in the 2–3 days range for mice and 4–5 days range for humans has the potential to cause a much stronger protection of the host. For example, the 75% reduction in blood IGF‐I caused by a 2‐ to 5‐day fast in mice and humans is not matched by CR, which causes a 25% IGF‐I reduction in mice and does not reduce IGF‐I levels in humans unless protein intake is also restricted. Even together with protein restriction, chronic CR only causes a 30% reduction in IGF‐I levels (reviewed in Lee & Longo, 2011).

In addition, we have noted a number of genes that were upregulated in the perilesional cortex of calorie‐restricted aged rats as compared to the perilesional area of *ad libitum*‐fed aged rats, including genes involved in glycogen metabolism, IGF signaling, apoptosis, arteriogenesis, and hypoxia. Downregulated genes in the perilesional cortex of the CR aged rats as compared to the perilesional area of *ad libitum*‐fed aged rats included genes involved in energy homeostasis (*Ins1, Igf1r, Mapk10),* apoptosis *(Camk2 g),* vasculogenesis *(Ppp2cb),* or tissue integrity (*Pmds6, Psmc4, Pmc3, Psmb5*).

In conclusion, our study shows that in poststroke, aged calorie‐restricted Sprague‐Dawley rats’ behavioral recuperation is enhanced as compared with *ad libitum*‐fed, overweighed rats. In this setting, there are an early gain in body weight and improved behavioral recovery that require complex sensorimotor skills, such as the rotating rod and inclined plane tasks, or cutaneous sensitivity and sensorimotor integration or spatial memory. Likewise, CR aged rats showed significant poststroke increases in serum glucose, insulin, and IGF1 levels as well as CR‐specific changes in gene expression including downregulation of genes involved in the ubiquitin proteasome degradation system (*Pmds6, Psmc4, Pmc3, Psmb5*), enhanced vasculogenesis (*Ppp2cb*), neuroprotection (*Mapk10*), and reduced apoptosis (*CaMKIIc*). All of these changes have been recorded in parallel with an increased expression of genes that are neuroprotective (*Igfbp3*), support angiogenesis (*Igf2*,* Mapkapk2*), and allow an increase in available energy (*Prkaac/Prkga1*). However, more experimental studies are needed, in order to fully understand the active complex interrelationships in this pathological setting.

## Experimental procedures

### Animals and experimental design

Young (3–4 months) and aged (19–20 months) male Sprague‐Dawley rats, bred in‐house, were used for our experiments. Previous research has reported that if fed *ad libitum* (AL), these animals develop a pattern of adipose deposition and obesity somewhat similar to humans. Remarkably, this type of obese rats will develop in time pancreatic pathological conditions such as fibrosis, islet hyperplasia, and exocrine atrophy as well mammary and pituitary tumors (Dillberger, 1994). The rats were randomly divided into the control group (young, *n* = 30; aged, AL, *n* = 30) and aged calorie‐restricted (CR) group (*n* = 30). Of these, three groups of rats (*n* = 7 each) were used to measure body fat mass in young, aged AL, and aged CR before surgery. Please note that the animal numbers refer to the subjects who survived 14 days after stroke. Body weights ranged from 290 to 360 g for the young rats and from 610 to 700 g for the aged rats at the time point when calorie restriction was initiated. The rats were kept in a temperature (22 °C)‐, humidity (40–60%)‐, and light period (07.00–19.00 h)‐controlled environment. The rats had free access to water.

#### Calorie restriction

The 20‐month‐old CR rats were fed on 70% of the average amount consumed by AL age‐matched rats, in a 2 days/week fasting regimen and in total for 8 weeks before stroke. The average amount of calories intake per day for the AL rats was of 79 kcal. The regular food pellet had the following composition: 65% carbohydrate, 29% protein, and 6% fat with a physiologic fuel value of 3.3 kcal/g pellet. Food administration was adjusted weekly, and the body weight was measured weekly based on *ad libitum* food intake of the previous week. Young rats were fed ad libitum. Drinking water was available at all times. The CR rats were also given a vitamin supplement.

##### Surgery

Eighteen hours prior to surgery, male Sprague‐Dawley rats were fasted but allowed free access to water to minimize variability in ischemic damage that can result from varying plasma glucose levels. All experiments were approved by the University Animal Experimentation Ethics Board according to the ethical requirements of the National Act on the Use of Experimental Animals and were in accordance with European Union directive.

##### Reversible occlusion of the middle cerebral artery

Blood flow through the middle cerebral artery was transiently interrupted in deeply anesthetized rats as previously described (Popa‐Wagner *et al*., 2010). The right middle cerebral artery was slowly lifted with a tungsten hook attached to a micromanipulator until blood flow through the vessel was completely stopped. Both common carotid arteries were then occluded by tightening prepositioned thread loops. The blood flow was monitored with a Laser Doppler (Periflux 5000; Perimed, Stockholm, Sweden) by positioning the optic tube on the temporal bone of rat skull. A decrease in the laser Doppler signal to <20% of control values was considered to indicate successful MCA occlusion. After 90 min, the middle cerebral artery and the common carotid arteries were re‐opened, allowing full reperfusion of the brain.

Following survival times of 14 days, the rats were deeply anesthetized and perfused with buffered saline followed by buffered, 4% freshly depolymerized paraformaldehyde. Subsequently, the brain was removed, postfixed in 4% buffered paraformaldehyde for 24 h, cryoprotected in 20% sucrose prepared in 10 mmol/L phosphate‐buffered saline, flash‐frozen in isopentane, and stored at −70 °C until sectioning.

For real‐time PCR, the brains (*N* = 7) were perfused with buffered saline, cut into 2‐mm slices that were dipped in TTC to allow visualization of the infarct core. This procedure allowed us to microdissect the periinfarcted area of cortex and the corresponding cortex area of contralateral healthy hemisphere that were then stored at −70 °C until use.

#### Behavioral analysis

Testing procedure involved two persons, one person who did the surgery and was in charge of handling the animals according to group assignment and another one who has tested the animals and was not aware of groups’ identity. To evaluate changes in neurological function associated with ischemia, the rats were subjected to a variety of locomotor, sensory, learning, and memory tests before and after surgery as previously described by our group (Buchhold *et al*., 2007; Popa‐Wagner *et al*., 2010). All testing was performed in the morning from 9 AM to 11 AM by the same investigator. Results obtained before surgery were used to define 100% functionality for each animal on each test, and functional recovery was expressed as percentage recovery relative to the presurgery baseline.

##### Beam‐walking test

The beam‐walking or *rotating rod* task assesses fine vestibule–motor function in the middle cerebral artery occlusion (MCAO) model (Buchhold *et al*., 2007). Each rat was tested for its ability to negotiate a rotating (6 rpm) horizontal rod.

##### Inclined plane

We tested the ability of each animal to maintain its position at a given angle on an inclined plane. The relative angle at which the rat could no longer maintain its position was taken as a measure of functional impairment. This test was conducted once before surgery and every other day thereafter.

##### Asymmetric sensorimotor deficit: adhesive Tape Removal Test

We assessed the asymmetry of sensory motor deficit of the forelimbs induced by unilateral MCAO by the adhesive tape removal test. Briefly, sticky patches were applied on the distal hairless parts of the forelimbs and the removal time from both limbs was measured. Three trials were performed separately for each limb, and the means of the values were noted. If the animal did not remove the tape within 180s, the timer was stopped. Results are given as ratio of time needed to remove the adhesive tape from one forelimb divided by the sum of time needed to remove it from both forelimbs (Popa‐Wagner *et al*., 2010).

##### Morris water maze

The Morris water maze task was used to assess spatial learning and memory. One week before surgery, aged rats were trained to find a submerged platform in a large (180 cm diameter) pool filled to within 20 cm of the upper edge with water maintained at 26 °C. The pool was divided into four compass quadrants (north, south, east, and west). Several visual stimuli were placed in each of the four quadrants. For the acquisition of spatial learning, each animal underwent a set of four trials per day for 7 days (Balseanu *et al*., 2014).

##### Determination of infarct volume by immunohistochemistry

To assess the size of the infarct induced by focal ischemia, we used anti‐NeuN immunostaining according to the protocol previously described (Popa‐Wagner *et al*., 2010). Every 20th, free‐floating section of 25 μm was immunostained for NeuN to cover the entire infarcted volume, which was then calculated as the sum of partial areas using the MicroBrightField (Colchester, VT, USA) system. Integration of the resulting partial volumes (partial areas × # sections x section thickness × section intervals) yielded the total volume of the ipsilateral hemisphere along with the total volume of the infarct.

#### Biochemical analysis

Fasting glucose was measured in blood obtained by retro‐orbital collection by directing the collection tube gently in a ventro‐lateral direction while rotating the tube. For multiple collection times, alternate right and left eyes were used. Kits were used to measure the concentrations of several metabolites in serum: insulin, IGF1 (IBL International, Hamburg, Germany), and free fatty acids (WAKO, Neuss, Germany).

#### Fat mass estimation

At the end of the caloric restriction period, rats were sacrificed (*N* = 7) and fat pads (mesenteric, retroperitoneal, epididymal, abdominal, and subcutaneous) were dissected, cleaned, and weighed. Fat mass was defined as the sum of the adipose pads that were dissected.

#### Gene expression analysis

##### RNA isolation

Total RNA was isolated from the microdissected tissue using TRIzol reagent (Invitrogen Life Technologies, Karslruhe, Germany) as described by the manufacturer, followed by DNase 1 (Ambion Kaufungen, Germany) digestion and further purified using RNeasy Mini extraction kit (Qiagen, Hilden, Germany). To avoid RNA degradation because of secondary hypoxia due to the microdissection procedure, we flash‐frozen the tissue immediately after microdissection. Purified total RNA was used for cDNA array assay and real‐time PCR quantification.

##### cDNA array assay

To analyze the effect of caloric restriction on recovery after stroke, we employed custom cDNA arrays containing 128 genes whose function is related to metabolic processes (SuperArray, Bethesda, MD, USA) according to the manufacturer's instructions. Data are presented as fold change, which is calculated as the ratio of experimental condition to control after normalization to housekeeping genes. The gene expression is given as fold change and was expressed as the mean of two experiments. Only those genes whose expression was equal to or greater than a twofold change were considered to be differentially regulated (Fig. 5). The list of genes is given under Supplementary files (Appendix S1).

After normalization to housekeeping genes, two sets of data for each age group, given as fold change, were generated: (i) We compared ipsilateral (periinfarct, PN) of AL rats vs. ipsilateral (periinfarct, PN) CR rats and (ii) contralateral (CL) of AL rats vs. contralateral (CL) of CR rats. Only those genes whose expression is equal to or more than 1.5‐fold change were considered as differentially regulated. For downregulated genes, the threshold was set to 0.5.

##### Quantitative real‐time PCR

For qualitative real‐time PCR (qPCR), cDNA was synthesized from total RNA with the High‐Capacity cDNA reverse transcription kit (Applied Biosystems, Foster City, CA, USA). All samples containing iQ SYBR Green Master Mix were amplified in triplicate. Data were analyzed using the ΔΔ*C*_t_ method. The expression levels of genes of interest were normalized to the average of expression level of the two housekeeping genes (hypoxanthine guanine phosphoribosyltransferase 1, HPRT1, and ribosomal protein 19, RPL 19). The fold change for a gene of interest was defined as the ratio of the relative expression in the ipsilateral hemisphere (periinfarct, PN) of AL rats to that in the ipsilateral hemisphere (periinfarct, PN) CR rats. All primers have been provided by Eurofins, Germany.

#### Statistics

The main effects of treatment and time as well as interactions of the two factors were analyzed using two‐way ANOVA with repeated measures (GraphPad Software, San Diego, CA, USA), with treatment as between‐subjects variable and time as within‐subjects variable. For quantitative data, the results were expressed as mean ± standard deviation (mean ± SD). The normality of the distribution of variables was determined by the Lilliefors test. The between‐groups analysis was performed using post hoc tests (Bonferroni) for multiple comparisons. The level of significance was set at *P* < 0.05, using two‐tailed test. Simple comparisons for normally distributed histological data were analyzed using Student's *t*‐tests. The level of significance was set at *P* < 0.05, using two‐tailed test.

## Conflict of interest

We have no conflict of interest to declare.

## Funding

No funding information provided.

## Author contributions

OC involved in calorie restriction, stroke model/surgery, behavioral experiments; body fat estimation; and manuscript writing. RES performed DNA arrays and biochemical analysis. ATB performed stroke model/surgery and perfusions. AZ performed behavioral experiments. AG performed stroke model/surgery. EB involved in data analysis and interpretation, and manuscript writing. AU performed RT–PCR. APW involved in conception and design, financial support, data analysis and interpretation, manuscript writing, and final approval of manuscript.

## Supporting information


**Appendix S1.** List of genes those expression is related to metabolism.
